# Ovarian Fibroma Mimicking an Endometrioma in a Postmenopausal Patient: A Diagnostic Challenge in a Solid Adnexal Mass With Low Risk of Ovarian Malignancy Algorithm (ROMA) Index

**DOI:** 10.7759/cureus.117684

**Published:** 2026-10-09

**Authors:** Vanessa García-Gudiño, Emiliana Arcos-Ayala, Santiago Córdova-Egüéz, Hugo Garzón-Ávila, Fedra Vela-Merino

**Affiliations:** 1 Department of Obstetrics and Gynecology, Hospital Metropolitano, Quito, ECU; 2 School of Medicine, Universidad San Francisco de Quito, Quito, ECU; 3 Department of Medical Education, Hospital Metropolitano, Quito, ECU

**Keywords:** adnexal mass, endometrioma, ovarian fibroma, postmenopause, roma index

## Abstract

Ovarian fibromas are uncommon benign sex cord-stromal tumors that frequently present as unilateral solid masses and may mimic ovarian malignancy, particularly in postmenopausal women. Preoperative diagnosis remains challenging because imaging findings and tumor markers often overlap with those of other adnexal lesions. We present the case of an ovarian fibroma initially misdiagnosed as an endometrioma, illustrating the limitations of preoperative assessment. We report the case of a 60-year-old postmenopausal woman with an incidentally detected right adnexal mass initially interpreted as an endometrioma on transvaginal ultrasound and computed tomography (CT). The patient subsequently underwent laparoscopic resection, and histopathological examination confirmed the diagnosis of ovarian fibroma. Ovarian fibromas may mimic other adnexal masses, making preoperative diagnosis challenging. Imaging studies and serum tumor markers have limited specificity and may be insufficient to reliably distinguish these tumors from other ovarian lesions. In postmenopausal women, persistent solid adnexal masses generally warrant surgical exploration regardless of ancillary findings. Therefore, definitive diagnosis of ovarian fibroma relies on histopathological examination. Ovarian fibroma remains an important differential diagnosis of solid adnexal masses in postmenopausal women. The limitations of preoperative assessment reinforce the role of surgical excision, while histopathological examination remains the gold standard for definitive diagnosis.

## Introduction

Among ovarian tumors, fibromas are the most common type of tumor arising from cells of the sex cord stroma. They are benign, relatively rare, and account for 4% of ovarian tumors. They can occur at any stage of a woman's life, with the highest prevalence (77%) during reproductive age [1]. They usually present unilaterally in approximately 90% of cases. Their size varies, with an average diameter of about 6 cm. They are characterized by slow growth, although they can reach considerable sizes [2].

Larger ovarian fibromas carry a substantially higher risk of complications, notably ascites and ovarian torsion, making tumor size a critical factor in clinical prognosis [3]. Clinical presentation is variable and covers a wide spectrum. While many patients are completely asymptomatic, others may present with non-specific lower abdominal or pelvic symptoms, including chronic pelvic pain, localized discomfort, abdominal distension, a palpable mass, or compressive symptoms affecting adjacent organs, such as urinary frequency or bowel habit changes. In other instances, the condition is discovered as an incidental finding in imaging studies, or it can present with complications such as ascites and pleural effusion, a clinical picture characteristic of Meigs syndrome [4].

The diagnostic workup for these tumors includes laboratory testing and imaging modalities to guide the diagnosis. On computed tomography (CT), these lesions typically present with variable attenuation, solid dense components, or degenerative cystic changes that closely mimic endometriomas or malignant lesions. However, definitive diagnosis is achieved exclusively through histopathology. As solid tumors of stromal origin, they are frequently accompanied by elevated CA-125 levels, particularly when associated with Meigs syndrome. Nonetheless, this marker can be elevated in benign conditions and even in 2% of healthy women. Therefore, its clinical utility remains controversial, and there is currently no specific biomarker for ovarian fibromas [5]. 

In this scenario, the evaluation of a solid adnexal mass in a postmenopausal patient remains a clinical challenge despite advancements in imaging and the use of tumor markers. Furthermore, preoperative differentiation between benign and malignant lesions continues to be limited, particularly regarding stromal tumors. Herein, we present the case of a postmenopausal patient with a solid adnexal mass whose initial imaging diagnosis suggested an endometrioma, but whose histopathological examination ultimately confirmed an ovarian fibroma.

## Case presentation

A 60-year-old postmenopausal woman (10 years postmenopause) with a medical history of Sjögren's syndrome, under occasional treatment with methotrexate and folic acid, presented for a routine gynecological evaluation after several years of lost follow-up. Past surgical history included a hysterectomy for abnormal uterine bleeding 20 years prior. Her obstetric history noted gravida 3, para 1, abortus 2, with one living child (G3P1A2). She was not currently sexually active. No other significant history was noted. Baseline laboratory exams showed mild leukopenia as the only remarkable finding.

The patient reported no active symptoms such as acute pelvic pain, abnormal bleeding, or systemic B-symptoms; her presentation was entirely asymptomatic. During this routine evaluation, a transvaginal ultrasound was performed, which incidentally revealed a solid mass in the right ovary measuring approximately 50 × 46 × 64 mm (volume: 67 mL), with no free fluid in the pouch of Douglas. No other abnormalities were detected. 

Based on the mentioned findings, a non-contrast and contrast-enhanced computed tomography (CT) scan of the abdomen and pelvis was requested (Figure 1). It revealed an ovoid-shaped mass in the right adnexa with cystic-dense content (20-45 HU) and well-defined margins showing mild contrast enhancement, measuring 57 × 41 mm in its largest diameters, compatible with an endometrioma. To further characterize the tumor behavior, serum tumor markers were ordered (Table 1). All results were within the reference ranges, and the Risk of Ovarian Malignancy Algorithm (ROMA) index indicated a low risk of malignancy. 

**Figure 1:**
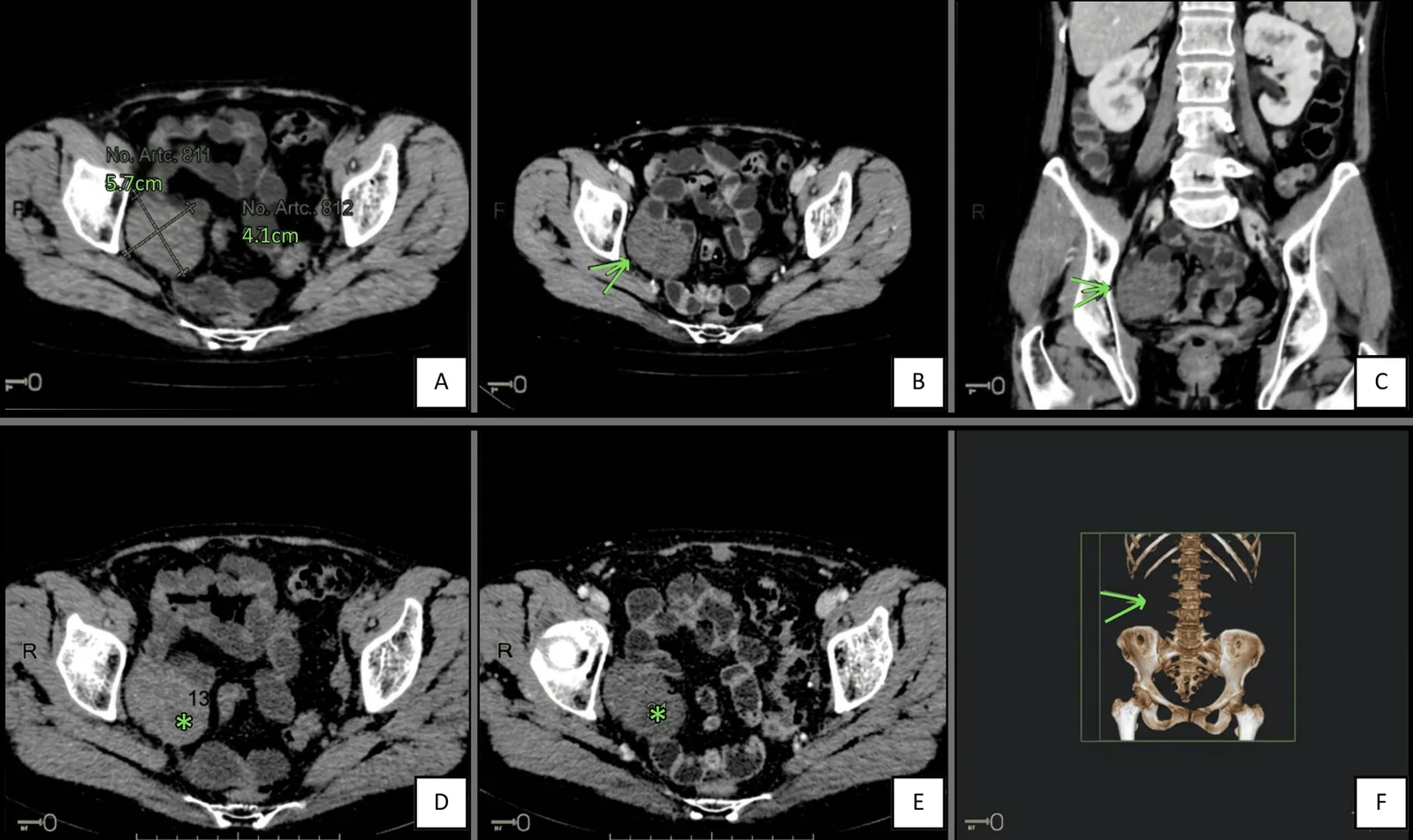
Non-contrast and contrast-enhanced abdominopelvic computed tomography (CT) showing a right adnexal mass. (A) Axial view showing a well-circumscribed right adnexal mass with maximal dimensions (5.7 cm × 4.1 cm at its greatest diameters). (B) Axial view showing an ovoid-shaped lesion with dense cystic content (20-45 HU) and well-defined borders after contrast administration. (C) Coronal reconstruction demonstrating the location of the right adnexal mass. (D) and (E) Additional axial images illustrating the relationship of the lesion with adjacent pelvic structures. (F) Three-dimensional reconstruction indicating the anatomical location of the mass.

**Table 1:** Preoperative serum tumor marker results and corresponding reference ranges. β-hCG: beta-human chorionic gonadotropin; ROMA: Risk of Ovarian Malignancy Algorithm.

Laboratory test	Patient value	Reference range
β-hCG	1.36 mIU/mL	<5.0 mIU/mL (non-pregnant)
Alpha-fetoprotein	4.33 µg/L	0-10 µg/L
CA-125	23.20 U/mL	<35 U/mL
CA 15-3	9.90 U/mL	<25 U/mL
HE4	65 pmol/L	<140 pmol/L (postmenopausal)
ROMA index	18.95%	Reference cutoff (postmenopausal): <27.7%

Given the presence of a persistent solid adnexal mass in a postmenopausal woman, surgical management via operative laparoscopy was decided. During the procedure, a solid right ovarian mass was observed, measuring approximately 6 cm in diameter, with a regular surface and a whitish appearance (Figure 2). A total surgical resection was performed. Gross pathological examination revealed multiple whitish fragments weighing 40 g in total, with a whirled, whitish cut surface (Figure 3). Microscopic analysis showed a proliferation of bland spindle cells with ovoid nuclei and scanty cytoplasm, accompanied by an extensively collagenized interstitium, without mitotic activity. No necrosis was identified (Figure 4). These findings were compatible with an ovarian fibroma. Finally, the patient had a satisfactory postoperative course without complications.

**Figure 2:**
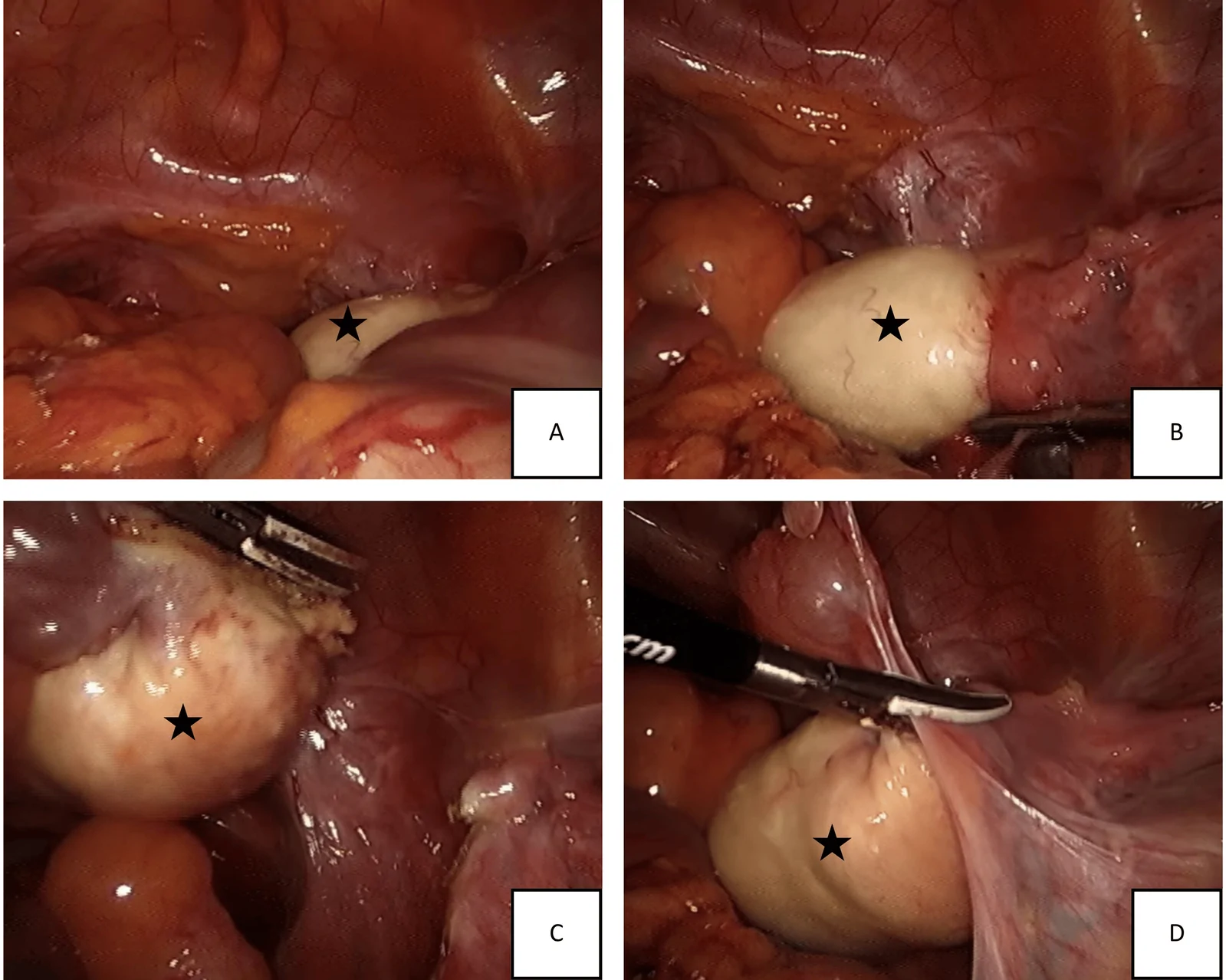
Intraoperative laparoscopic findings. (A) and (B) A solid mass (*) arising from the right ovary is observed, well-circumscribed relative to adjacent structures. It features a smooth surface, pearly appearance, and firm consistency. (C) and (D) Protocol-based oophorectomy, initiated at the infundibulopelvic ligament using advanced bipolar energy.

**Figure 3:**
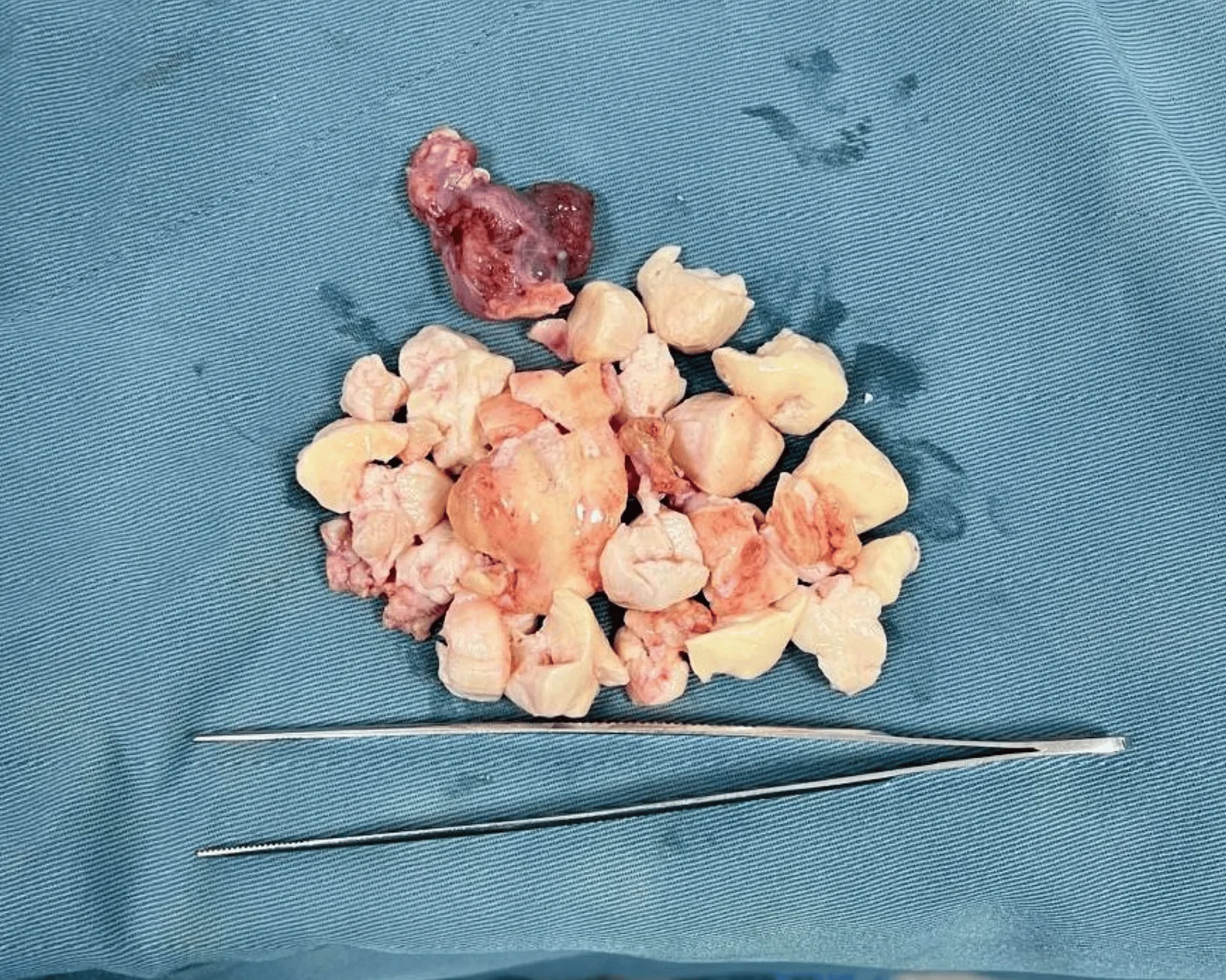
Gross anatomy of the resected right ovarian mass demonstrating multiple fragmented tissue specimens. The sectioned surfaces exhibit a characteristic firm, whirled, grayish-white solid appearance typical of an ovarian fibroma. No obvious areas of hemorrhage or macroscopic cystic necrosis were evidenced.

**Figure 4:**
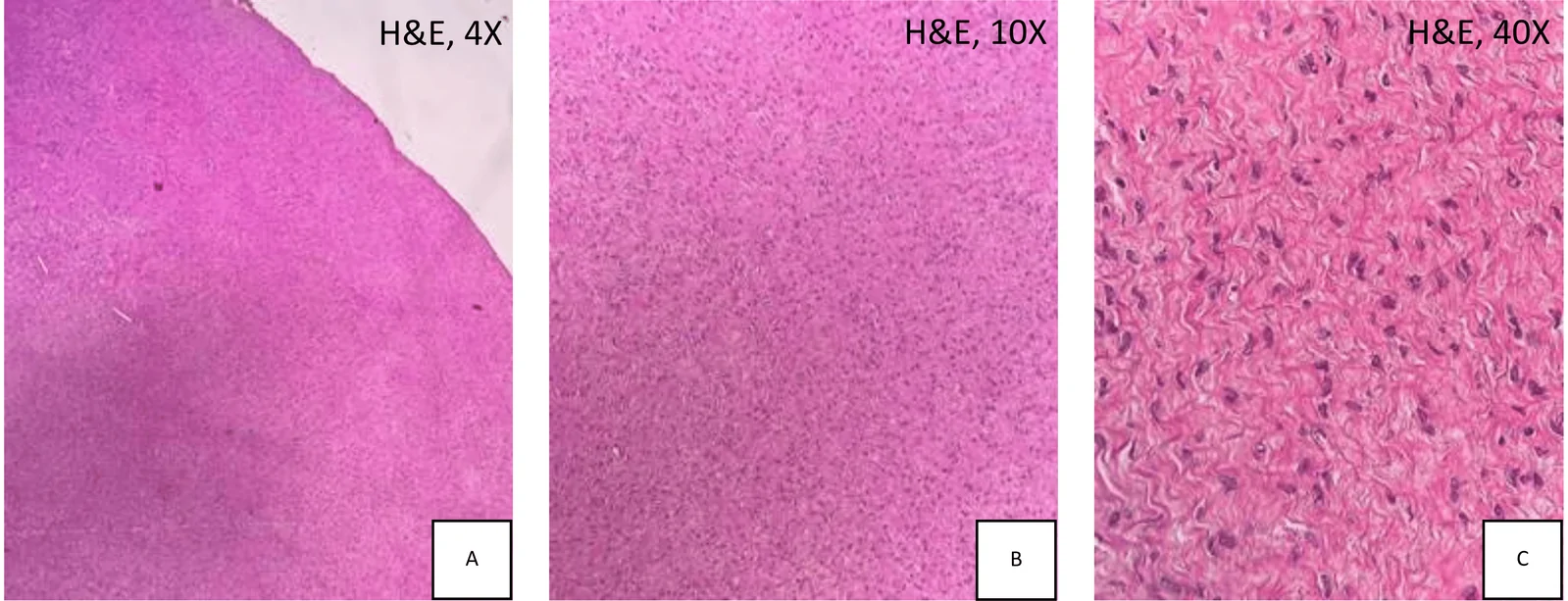
Histopathological study of the ovarian tumor with hematoxylin and eosin (H&E) staining. (A) Low-power panoramic view (4×) showing a well-circumscribed solid lesion. (B) Intermediate magnification (10×) demonstrating a diffuse proliferation of spindle cells arranged in short fascicles. (C) High-power magnification (40×) showing spindle cells with ovoid nuclei, bland cytology, without significant atypia or mitotic activity, embedded in an abundant collagenous stroma. These findings are compatible with an ovarian fibroma.

## Discussion

Although ovarian fibroma represents the most prevalent benign sex cord-stromal tumor [6], its manifestation as a unilateral solid mass in postmenopausal females can pose a diagnostic dilemma, as any solid tumor within this cohort must be presumed potentially malignant until proven otherwise [7]. While transvaginal ultrasonography serves as the first-line diagnostic modality for evaluating ovarian masses [8], its findings can frequently overlap with other adnexal entities. Thus, computed tomography (CT) constitutes a valuable adjunctive tool [9].

Nonetheless, in the presented case, the lesion was initially categorized as a suspected endometrioma, an entity that is exceptionally rare in postmenopausal females [10]. Confirmatory histopathology revealed an ovarian fibroma, underscoring the suboptimal specificity of imaging modalities in characterizing stromal tumors. Current evidence demonstrates that computed tomography (CT) exhibits limited diagnostic performance and can mislead the preoperative characterization of adnexal masses. Consequently, its utility as a first-line tool for initial evaluation is not supported [11]. 

Moreover, MRI plays a fundamental role in evaluating ovarian tumors, enabling patient stratification into low- or high-risk categories for malignancy, with a reported specificity of approximately 76% [12]. Key diagnostic benefits of MRI rely on structural tissue characterization; findings indicative of ovarian fibromas/thecomas include characteristic hypointensity on T2-weighted imaging, specific diffusion coefficients, and moderate enhancement profiles [13]. Nevertheless, this study was omitted in our patient. Although MRI improves diagnostic precision in selected cases, factors such as cost and accessibility limited its use, justifying immediate surgical resolution. Consequently, performing an MRI would not have modified the clinical decision to pursue prompt surgical exploration.

On the other hand, serum tumor biomarkers and the ROMA index indicated a low risk of malignancy. However, it is critical to note that the ROMA index has been validated primarily for epithelial ovarian tumors; consequently, its predictive value for sex cord-stromal tumors remains limited [14]. While the absence of ascites and elevated CA-125 levels effectively ruled out Meigs syndrome, it did not entirely exclude malignancy. Stromal neoplasms can present with elevated CA-125, yet this biomarker may also rise in benign conditions and in up to 2% of healthy individuals [15]. Therefore, its diagnostic utility is controversial, and no specific biomarker currently exists for ovarian fibromas. Furthermore, current clinical management guidelines dictate that a persistent solid adnexal mass in a postmenopausal female warrants surgical evaluation regardless of benign ancillary imaging or biomarker findings, given the significantly elevated baseline risk of malignancy within this age group [11,16,17]. Although the initial radiological impression suggested an endometrioma, surgical exploration was pursued to mitigate potential complications. Ultimately, this surgical approach enabled definitive diagnostic confirmation via histopathology, which remains the gold standard for these lesions. This case highlights an important diagnostic triad in postmenopausal women--a persistent solid adnexal mass, low ROMA index/normal tumor markers, and misleading imaging characteristics, such as CT density mimicking an endometrioma. Identified together, these features emphasize that low-risk markers should not rule out surgical exploration when solid adnexal architecture is present.

## Conclusions

Ovarian fibroma is the most prevalent benign sex cord-stromal tumor and must be included in the differential diagnosis of any solid adnexal mass, particularly in postmenopausal females. Current evidence supports a comprehensive diagnostic approach integrating clinical findings, imaging modalities, and tumor biomarkers for accurate risk stratification. Notably, in postmenopausal patients, persistent solid lesions generally warrant surgical excision, which provides both therapeutic resolution and definitive diagnostic confirmation via histopathology--the gold standard for these tumors--while mitigating potentially life-threatening complications. This case underscores the clinical imperative of maintaining a high index of suspicion and implementing a systematic approach to ensure timely and safe management aligned with current guidelines, without disregarding benign etiologies in clinical scenarios highly suggestive of malignancy.
